# Aminoglycosides, but not PTC124 (Ataluren), rescue nonsense mutations in the leptin receptor and in luciferase reporter genes

**DOI:** 10.1038/s41598-017-01093-9

**Published:** 2017-04-21

**Authors:** Florian Bolze, Sabine Mocek, Anika Zimmermann, Martin Klingenspor

**Affiliations:** 1 ZIEL - Institute for Food and Health, Technical University of Munich, Gregor-Mendel-Str. 2, 85354 Freising, Germany; 2 Chair of Molecular Nutritional Medicine, Technical University of Munich, EKFZ - Else Kröner-Fresenius-Center for Nutritional Medicine, Gregor-Mendel-Str. 2, 85354 Freising, Germany

## Abstract

In rare cases, monogenetic obesity is caused by nonsense mutations in genes regulating energy balance. A key factor herein is the leptin receptor. Here, we focus on leptin receptor nonsense variants causing obesity, namely the human W31X, murine Y333X and rat Y763X mutations, and explored their susceptibilities to aminoglycoside and PTC124 mediated translational read-through *in vitro*. In a luciferase based assay, all mutations - when analysed within the mouse receptor - were prone to aminoglycoside mediated nonsense suppression with the highest susceptibility for W31X, followed by Y763X and Y333X. For the latter, the corresponding rodent models appear valuable for *in vivo* experiments. When W31X was studied in the human receptor, its superior read-through susceptibility – initially observed in the mouse receptor – was eliminated, likely due to the different nucleotide context surrounding the mutation in the two orthologues. The impact of the surrounding context on the read-through opens the possibility to discover novel sequence elements influencing nonsense suppression. As an alternative to toxic aminoglycosides, PTC124 was indicated as a superior nonsense suppressor but inconsistent data concerning its read-through activity are reported. PTC124 failed to rescue W31X as well as different nonsense mutated luciferase reporters, thus, challenging its ability to induce translational read-through.

## Introduction

Nonsense mutations are single nucleotide exchanges that cause in frame premature termination codons (PTCs), thus, leading to the synthesis of truncated and dysfunctional proteins. The impact of nonsense mutations on human health is indicated by many inherited diseases, such as cystic fibrosis (CF) and Duchenne muscle dystrophy (DMD)^1, 2^. In rare cases, human monogenetic obesity can be caused by PTCs in genes encoding for leptin^3^ and its cognate receptor (LEPR)^4, 5^, pro-opiomelanocortin^6^, prohormone convertase 1^7^ and melanocortin-4-receptor^8^, which are all engaged in central energy balance regulation.

Aminoglycoside antibiotics have the ability to suppress translation termination at PTCs^9^. The reduction of translation fidelity permits the pairing of a near-cognate aminoacyl-tRNA with the PTC and thereby allows the continuation of protein synthesis^10^. The read-through efficiency depends on the nature of the PTC (TGA > TAG > TAA) and the surrounding nucleotide context^11–13^. A multitude of preclinical studies have emphasized nonsense suppression as a strategy to treat inherited diseases^14–17^. However, clinical studies resulted in variable outcomes: only subpopulations of patients suffering nonsense mutation CF, DMD, McArdle disease, or haemophilia benefit from aminoglycoside treatment^18–21^. Moreover, the use of aminoglycosides is limited due to side effects^22, 23^. Alternative drugs are needed to enable an efficient and safe nonsense suppression therapy. In several preclinical studies^24–31^, the small molecule PTC124 (Ataluren®) was suggested as the desired drug with superior suppressor properties and mild side effects^32^. In addition, a Phase 2a and a subsequent Phase 2b study conducted with patients suffering nonsense mutation DMD demonstrated that PTC124 elevates dystrophin levels in muscle biopsies and slightly reduces the disease progression relative to placebo^33, 34^. In 2014 - distributed under Translarna - PTC124 received a ‘conditional approval’ from the European Medicines Agency for DMD^35^. Noteworthy, several preclinical reports could not confirm the read-through activity of PTC124^36–41^ and the clinical efficiency – in particular for CF patients in a Phase 3 study^42^ - was evaluated as rather weak^43^.

Only a few studies have addressed the suppression of PTCs in obesity-related genes^36, 44^. In the present work we focus on the leptin receptor, a class I cytokine receptor strongly expressed in the hypothalamus. Plasma levels of the adipocyte-derived hormone leptin communicate the stage of energy storage to the central nervous system^45^. The plasma membrane bound LEPR-b isoform has a molecular weight of 132 kD and signals through diverse pathways including the Janus kinase/signal transducer and activator of transcription 3 (JAK/STAT3) cascade^46^. The importance of leptin and its receptor on energy balance is highlighted by loss-of-function mutations causing rare forms of monogenic obesity^4, 47^. Recombinant leptin represents a successful therapy to treat congenital leptin deficiency^48, 49^. Restoring leptin receptor expression in the brain is also a beneficial treatment but its implementation is more difficult than leptin replacement therapy^50^.

Here, we studied three leptin receptor nonsense mutations, as well as their suppression susceptibility to the aminoglycosides G418 and gentamicin, and to the oxadiazole substance PTC124 in HEK293 cells. *LEPR* ^*W31X*^ was originally found in an obese human subject^4^, *Lepr* ^*Y333X*^ was identified in the obese *db*_*333*_/*db*_*333*_ mouse model^51^, and *Lepr* ^*Y763X*^ represents the mutation causing the obese phenotype of Koletsky rats^52^. To ensure comparability between the variants originating from different species, we initially characterized them within the murine receptor. The W31X variant was additionally investigated in its natural human receptor context. Firstly, we conducted an assay with fusion constructs consisting of the particular PTC ± 6 bp nucleotide context and a *Photinus* luciferase (PLuc) to assess the read-though susceptibility of the mutations. Then we performed a signalling assay utilizing STAT3-responsive luciferase reporter genes to investigate the receptor activities of the LEPR-b mutants. Additionally, we designed and tested nonsense mutated *Renilla* and secreted *NanoLuc* luciferase reporters to further explore the read-through activity of PTC124.

## Results

### Susceptibility of nonsense mutated mLepr-PLuc fusions constructs to aminoglycoside mediated read-through

**Figure 1:**
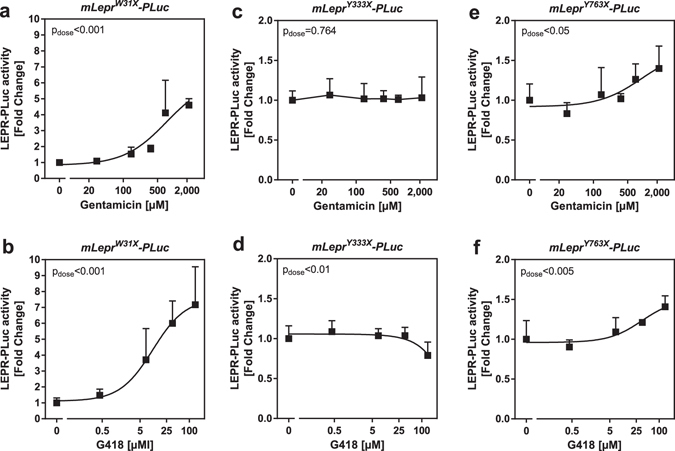
Susceptibility of nonsense mutated *Lepr*-*PLuc* fusion constructs to aminoglycoside mediated read-through. HEK293 cells were co-transfected with either (**a**,**b**) *mLepr* ^*W31X*^-*PLuc*, (**c**,**d**) *mLepr* ^*X333Y*^-*PLuc* or (**e**,**f**) *mLepr* ^*X763Y*^-*PLuc* and phRG-b for data normalization. The indicated nonsense mutations were imbedded into ± 6 bp murine receptor context (see Table 1 and Supplementary Fig. 1). Two days after the transfection, cells were treated for 24 h with rising concentrations of either G418 or gentamicin (n = 4–6, SD).

All experiments were performed in medium free of streptomycin, an aminoglycoside antibiotic which is widely used in combination with penicillin in mammalian cell cultures. Pilot experiments have shown that the absence of streptomycin increased the read-through efficiency of gentamicin (Supplementary Fig. 2). Under this optimized cell culture condition, we tested whether W31X, Y333X and Y763X are prone to aminoglycoside mediated read-through using fusion constructs containing the particular PTC ± 6 bp context fused inframe upstream to the *Photinus* luciferase (PLuc) ORF. Initially, the mutations were investigated in the mouse receptor context (Table 1). Transiently transfected HEK293 cells were incubated for 24 h with rising concentrations of gentamicin or G418. Gentamicin rescued *mLepr* ^*W31X*^-*PLuc* (Fig. 1a), but less efficient than G418 since higher doses were needed and maximal luciferase activities were lower (Fig. 1b). On the contrary, both aminoglycosides failed to induce a read-through in *mLepr* ^*Y333X*^-*PLuc* transfected cells (Fig. 1c and d). In cells expressing m*Lepr* ^*Y763X*^-*PLuc*, gentamicin and G418 mediated only a minor restoration of luciferase activity (Fig. 1e and f).

**Table 1:** Leptin receptor nonsense mutations.

Nonsense mutation	Species	PTC ± 6 bp context	Protein
W16X	human*	ACT CCT **TGA** AGA TTT	TP**X**RF
	mouse	TCT CCC **TGA** AAA TTT	SP**X** KF
Y333X	mouse	GTT GTG **TAA** TTT CCA	VV**X**FP
Y763X	rat*	AAT GAT **TAA** AGT CTG	ND**X**SL
	mouse	GAT GAT **TAA** AGT CTG	DD**X**SL

Three different point mutations resulting in the formation of premature termination codons (PTC) were investigated in the present study. All mutations were inserted into the murine receptor sequence to ensure a comparison within an identical genetic backbone. The mutation W31X was additionally investigated within the human sequence. Differences on the nucleotide and amino acid level are underlined.

*Indicates the species in which the mutation was identified.

### Aminoglycoside mediated rescue of nonsense mutated mLEPR-b variants

**Figure 2:**
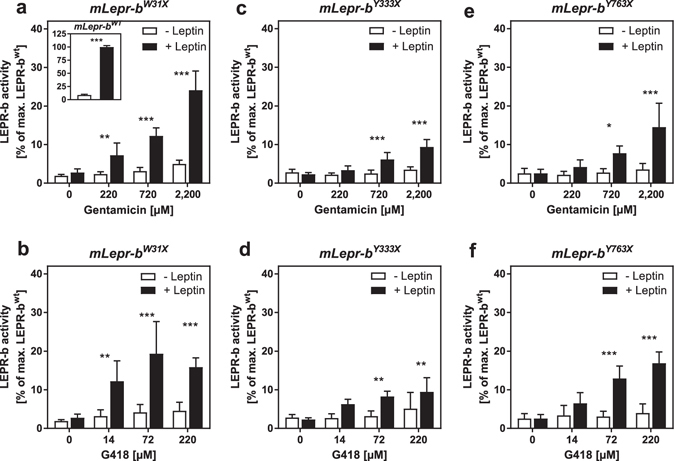
Aminoglycoside mediated rescue of signalling properties of nonsense mutated LEPR-b variants. HEK293 cells were co-transfected with either (**a**,**b**) *mLepr*-*b* ^*W31X*^- (inset *mLepr*-*b* ^*wt*^), (**c**,**d**) *mLepr*-*b* ^*Y333X*^- *or* (**e**,**f**) *mLepr*-*b* ^*Y763X*^-pcDNA3.1 overexpression construct, STAT3-RE-PLuc and phRG-b. Two days after the transfection, cells were treated for 24 h with different concentrations of the aminoglycosides and for the last 16 h in addition with 6 nM murine leptin (n = 4, SD). Statistical significance between (−) and (+) leptin was assessed by t-tests with Bonferroni-Holm correction for multiple comparisons *p < 0.05; **p < 0.01; ***p < 0.001.

In this assay, we tested whether aminoglycosides are able to restore the signalling properties of PTC harbouring full-length receptor variants. The nonsense variants *mLepr*-*b* ^*W31X*^, *mLepr*-*b* ^*Y333X*^ and *mLepr*-*b* ^*Y763X*^ were initially characterized within the mouse receptor ORF. Contrary to the wild-type receptor (Fig. 2a inset), in the absence of aminoglycosides none of the mutated variants were activated by leptin (Fig. 2). In line with the findings obtained with the PTC-containing *Lepr*-*PLuc* fusion vectors (Fig. 1), the most efficient rescue was detected for mLEPR-b^W31X^ (Fig. 2a and b), followed by mLEPR-b^Y763X^ (Fig. 2c and d) and mLEPR-b^Y333X^ (Fig. 2e and f). Both aminoglycosides were able to revive mLEPR-b^W31X^ signalling activity to 20–25% of the level of mLEPR-b^wt^ (Fig. 2a and b). Relative to mLEPR-b^wt^, mLEPR-b^Y333X^ (Fig. 2c and d) and mLEPR-b^Y763X^ (Fig. 2e and f) reached activity levels of 10% and 15%, respectively. Furthermore, G418 was more efficient than gentamicin since much lower doses were sufficient to induce receptor activity.

### PTP1B neutralizes the gentamicin induced rescue of mLEPR-b^W31X^ signalling

**Figure 3:**
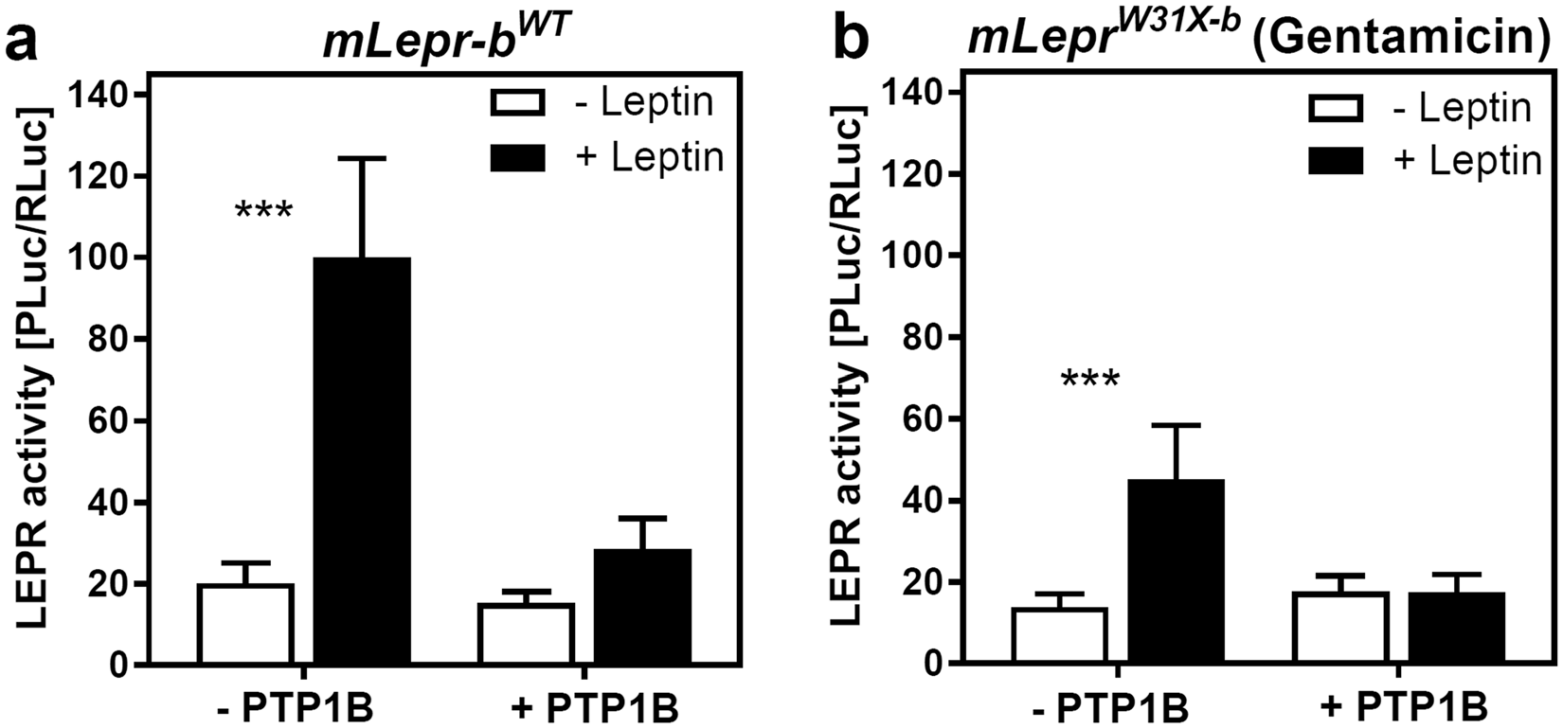
PTB1B blocks the gentamicin induced rescue of mLEPR-b^W31X^ signalling. HEK293 cells were co-transfected with either (**a**) *mLepr*-*b* ^*wt*^- or (**b**) *mLepr*-*b* ^*W31X*^-pcDNA3.1 overexpression construct, the PTB1B overexpression vector, STAT3-RE-PLuc and phRG-b. Two days after the transfection, cells were treated for 24 h with 2.2 mM gentamicin and for the last 16 h in addition with 6 nM murine leptin (n = 4, SD). Statistical significance between (−) and (+) leptin was assessed by t-tests with Bonferroni-Holm correction for multiple comparisons ***p < 0.001.

Protein-tyrosine phosphatase 1B (PTP1B) is an intracellular inhibitor of LEPR-b signalling. Its expression attenuated the activity of mLEPR-b^wt^ (Fig. 3a). Overexpression of PTP1B neutralized the gentamicin effect on mLEPR-b^W31X^ (Fig. 3b) indicating that a reactivation of the signalling cascade and not an off-target effect is responsible for the aminoglycoside mediated activation of the STAT3-RE-PLuc reporter in the signalling assay (Fig. 2).

### Aminoglycosides do not restore hLEPR-b^W31X^ signalling

**Figure 4:**
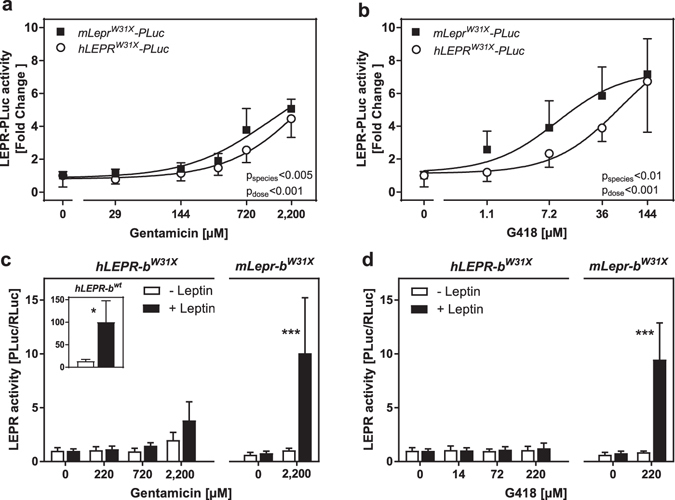
Aminoglycoside treatment of human LEPR^W31X^. (**a**,**b**) HEK293 cells were co-transfected with the *mLepr* ^*W31X*-^ *PLuc* or *hLEPR* ^*W31X*-^ *PLuc* fusion construct (embedding the W31X either into mouse or human ± 6 bp context) and phRG-b. Two days after the transfection, cells were treated for 24 h with rising concentrations of (**a**) gentamicin or (**b**) G418 (n = 7–8, SD). P-values indicate the outcome of two-way ANOVA with aminoglycoside treatment and species context as independent variables. (**c**,**d**) HEK293 cells were co-transfected with either the *hLEPR*-*b* ^*W31X*^-pDEST26 or *mLepr*-*b* ^*W31X*^-pcDNA3.1 full-length overexpression construct, STAT3-RE-PLuc and phRG-b. One day after the transfection, cells were treated for 48 h with different concentrations of the aminoglycosides gentamicin (**c**) or G418 (**d**) and for the last 16 h in addition with 16 nM human or 6 nM murine leptin, respectively. Inset in (**c**) shows signalling of *hLEPR*-*b* ^*wt*^ (n = 4–6, SD). Statistical significance was assessed by t-tests with Bonferroni-Holm correction for multiple comparisons *p < 0.05; ***p < 0.001.

For a more physiological characterization of the human mutation W31X, we studied the mutation in its natural human sequence context. When HEK293 cells - expressing the human or mouse LEPR^W31X^-PLuc fusion protein - were exposed to gentamicin and G418, a significantly lower read-through susceptibility for the human version was observed (Fig. 4a and b). Then, we compared the signalling properties of human and murine full-length LEPR-b^W31X^ orthologues. HEK293 cells transfected with the *hLEPR*-*b* ^*wt*^-pDEST26 expression construct were activated by leptin proving the functionality of the expression vector/system (Fig 4c inset). Signalling of hLEPR-b^W31X^ could not be restored in response to 24 h aminoglycoside treatment (data not shown). Even a prolonged G418 incubation of 48 h had no effect on hLEPR-b^W31X^ signalling (Fig. 4d). Only a modest activation of hLEPR-b^W31X^ was observed when cells were exposed for 48 h to the highest gentamicin concentration (Fig 4c). However, the difference between (−) and (+) leptin at 2200 µM gentamicin was not statistically significant. A two-way ANOVA conducted with the independent variables gentamicin and leptin treatment revealed a significant effect for both factors on hLEPR-b^W31X^ activity (gentamicin p < 0.001; leptin p < 0.05). In contrast, cells transfected with *mLepr*-*b* ^*W31X*^-pcDNA3.1 exhibited again a superior susceptibility to the read-through activity of both aminoglycosides (Fig. 4c and d).

The human and mouse LEPR-b orthologues were either expressed from pDEST26 or pcDNA3.1. To exclude a distracting effect of the pDEST26 vector backbone on nonsense suppression of W31X in the human receptor, we transferred the *hLEPR*-*b* ORF into pcDNA3.1. Replication of the experiment with *hLEPR*-*b* ^*W31X*^-pcDNA3.1 resulted again in no reactivation when cells were exposed to aminoglycosides (Supplementary Fig. 3).

### Off-target effect of PTC124 is specific for PLuc

**Figure 5:**
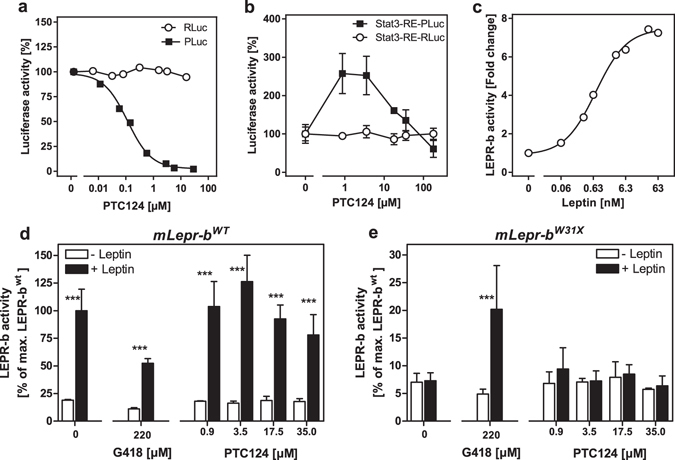
PTC124 has an off-target effect on PLuc and is not restoring mLEPR-b^W31X^ signalling. (**a**) Lysates from HEK293 cells containing PLuc and RLuc were treated with rising concentrations of PTC124 during bioluminescence quantification. Shown is one representative measurement. (**b**) HEK293 cells were co-transfected with the STAT3-RE-PLuc and STAT3-RE-Rluc vector. Two days after the transfection, cells were treated for 24 h with rising concentrations of PTC124 (n = 3, SD). (**b**) HEK293 cells were co-transfected with the *mLepr*-*b* ^*wt*^ overexpression construct and STAT3-RE-RLuc. Two days after the transfection, cells were treated for 16 h with rising concentrations of murine leptin. Shown is one representative measurement. (**d**,**e**) HEK293 cells were co-transfected with either the *mLepr*-*b* ^*wt*^- or *mLepr*-*b* ^*W31X*^-pcDNA3.1 overexpression construct and with STAT3-RE-RLuc. Two days after the transfection, cells were treated for 24 h with G418 or with rising concentrations of PTC124 and for the last 16 h in addition with 6 nM murine leptin (n = 3, SD). Statistical significance between (−) and (+) leptin was assessed by t-tests with Bonferroni-Holm correction for multiple comparisons ***p < 0.001.

Previous reports demonstrated off-target effects of PTC124 on PLuc, hence, disabling the STAT3-RE-PLuc reporter vector to study PTC124^38, 39^. Indeed, off-target effects of PTC124 on PLuc were confirmed in our study. When PTC124 was mixed with lysates from HEK293 cells containing PLuc and RLuc protein, PTC124 specifically reduced PLuc mediated bioluminescence in a dose-dependent manner but left the RLuc signal unperturbed (Fig. 5a). Intermediate PTC124 concentrations added to growing HEK293 cells, co-transfected with STAT3-RE-PLuc and STAT3-RE-RLuc vectors, increased PLuc, but not RLuc activity (Fig. 5b). At higher doses PLuc activity was reduced, most likely due to a carryover of PTC124 from the cell culture dish to the luminometer tube (Fig. 5b). Beside its desired insensitivity to PTC124, the STAT3-RE-RLuc vector was able to report LEPR-b signalling (Fig. 5c). The EC_50_ value assessed with the STAT3-RE-RLuc plasmid (1.1 nM) matches the EC_50_ value determined in previous experiments utilizing the STAT3-RE-PLuc reporter (0.8 nM)^53^.

### PTC124 does not rescue mLEPR-b^W31X^ signalling

The effect of PTC124 on LEPR-b signalling was investigated with the newly designed STAT3-RE-RLuc reporter vector. Under all treatment conditions, mLEPR-b^wt^ signalling was activated by leptin (Fig. 5d). The reduced mLEPR-b^wt^ activity in response to G418 is likely due to toxic side effects. Mouse LEPR-b^W31X^ activity was successfully reactivated in medium supplemented with G418. Notably, mLEPR-b^W31X^ signalling reached ~20% of the mLEPR-b^wt^ level (Fig. 5e), an effect size which is in line with the rescue effect assessed with the STAT3-RE-PLuc reporter construct (Fig. 2a). On the contrary, PTC124 did not restore mLEPR-b^W31X^ signalling at any dose applied (Fig. 5e).

### Aminoglycosides, but not PTC124, suppress nonsense mutations in the Renilla and secNLuc luciferase genes

**Figure 6:**
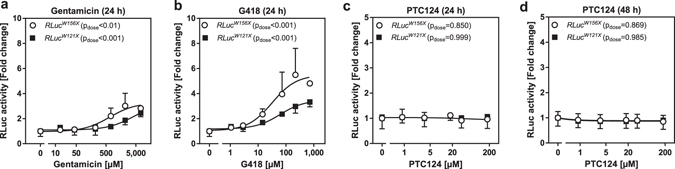
Aminoglycosides but not PTC124 suppress nonsense mutations in the *Renilla* luciferase. HEK293 cells were transfected with either the *RLuc* ^*W121X*^ or *RLuc* ^*W156X*^ overexpression construct. Two days after the transfection, cells were treated for 24 h with (**a**) G418, (**b**) gentamicin, (**c**) 24 h or (**d**) 48 h with PTC124 (n = 3–4, SD). Statistical significances were tested by one-way ANOVA with compound concentration as independent variable.

**Figure 7:**
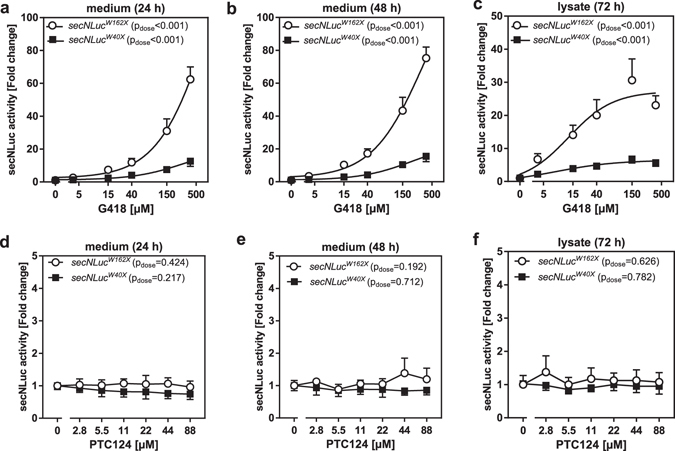
G418 but not PTC124 suppresses nonsense mutations in the secreted NanoLuc luciferase. HEK293 cells were transfected with either the *secNLuc* ^*W40X*^ and *secNLuc* ^*W162X*^ overexpression construct. Two days after the transfection, cells were treated for 24 h, 48 h and 72 h with rising concentrations of G418 (**a**–**c**) or PTC124 (**d**–**f**) (n = 4, SD). Luciferase activities were measured either in medium or in cell lysates.

To further study PTC124, we generated nonsense mutated *Renilla* and *secNLuc* luciferase reporter gene vectors. Gentamicin and G418 were able to rescue RLuc^W121X^ and RLuc^W156X^ activities in a dose-dependent manner after an incubation time of 24 h (Fig. 6a and b). In contrast, PTC124 was not able to revive the *Renilla* mutants even after a prolonged incubation of 48 h (Fig. 6c and d). Besides, we utilized secNLuc - a small luciferase which is secreted into the medium^54^ - as reporter for nonsense suppression. In the first place, we tested whether G418 and PTC124 have an off-target effect on secNLuc. We detected slight but tolerable off-target effects (during luminometry) and evaluated that secNLuc represent a suitable reporter system to assess the read-through activities of our test compounds (Supplementary Figures 4 and 5). G418 restored secNLuc^W40X^ and secNLuc^W162X^ activities dose-dependently in medium after 24 and 48 h as well as in cell lysates after 72 h incubation (Fig. 7a–c). Again, PTC124 failed to rescue the activity of secNLuc mutants at any treatment condition (Fig. 7d–f).

## Discussion

Approximately 10% of genetic diseases are driven by nonsense mutations^55^. In rare cases, monogenic obesity can also be the consequence of nonsense mutations in genes which are involved in body weight regulation.

In HEK293 cells, we characterized the human W31X^4^, murine Y333X^51^ and rat Y763X^52^ leptin receptor nonsense variants and their suppression susceptibilities to the aminoglycosides G418 and gentamicin and to the oxadiazole substance PTC124. For establishing a read-through assay, we cloned reporter gene constructs consisting of the particular PTC ± 6 bp murine nucleotide context fused to the N-terminus of the *Photinus* luciferase (PLuc). The highest read-through susceptibility was observed for *Lepr* ^*W31X*^-*PLuc*, followed by *Lepr* ^*Y763X*^-*PLuc*. The *Lepr* ^*Y333X*^-*PLuc* fusion did not respond to the aminoglycoside treatment. These findings are in general agreement with previous studies which demonstrated variable read-through levels dependent on the nature of the PTC (TGA > TAG > TAA) and the surrounding nucleotide context^11–13^.

For the signalling assay, we introduced the mutations into the full-length mouse *Lepr*-*b* ORF. The *Lepr*-*b* ^*W31X*^ allele exhibited again the highest rescue susceptibility followed by *Lepr*-*b* ^*Y763X*^. The increased bioluminescence during the aminoglycoside treatment was clearly caused by an activation of the JAK/STAT pathway since PTP1B blocked the gentamicin induced restoration. Cytoplasmic PTP1B dephosphorylates JAK2, attenuates STAT3 phosphorylation and thereby inhibits the production of PLuc^56^. Although, the read-through experiments with the *Lepr* ^*Y333X*^-*PLuc* fusion construct indicated a resistance of Y333X to aminoglycoside treatment, the signalling assay, however, revealed that Y333X is receptive for nonsense suppression. This inconsistency is likely due to the different sensitivities of the two assay systems. In the signalling assay, translational read-through is *indirectly* detected by measuring the complete signalling cascade with the STAT3-responsive reporter. Intracellular receptor pathways typically amplify the initial signal which is likely to potentiate the sensitivity of the assay. In the read-through assay, nonsense suppression is *directly* detected without any amplification cascade between the translational read-through event and the bioluminescence measurement. Moreover, the different length/complexities of the nucleotide contexts surrounding Y333X either in the fusion construct or in the full-length *Lepr*-*b* ORF may also contribute to the inconsistent results between the two assays.

The successful rescue of Y333X and Y763X identifies the *db*_*333*_/*db*_*333*_ mouse and the Koletsky rat as rodent models to explore nonsense suppression *in vivo*. Additionally, other strategies to rescue PTCs including inhibition of nonsense mediated mRNA decay, suppressor tRNAs and pseudourydilation can be explored in these two rodent models^57^. The success of pharmacological interventions depends strongly on the pharmacokinetic characteristics of a drug. A specific challenge for the rescue of nonsense mutations in centrally expressed genes is the blood-brain-barrier – a border which limits the delivery of drugs from the lumen of cerebral blood capillaries into brain parenchyma. Obstacles regarding blood-to-brain transfer as well as complication due to cytotoxocities must be considered in studies using animal models for nonsense suppression.

Protein identity for human/mouse and rat/mouse LEPR-b are 75% and 82%, respectively. The triplets encoding W31 and Y763, as well as the important +1 nucleotides are also conserved. Therefore, we initially examined all mutations in the murine sequence to compare their characteristics within the same context. For a more physiological characterization, we additionally investigated W31X within its natural human context. Interestingly, the superior read-through susceptibility of W31X disappeared when integrated in the full-length *hLEPR*-*b*. This ‘loss-of-response’ was already indicated in the read-through assay with the fusion constructs. Additional nucleotides outside of the ±6 bp context – which are only present in the full-length *hLEPR*-*b* ^*W31X*^ – could further reduce the read-through susceptibility. Alternatively, a full-length protein synthesis is not necessarily synonymous to the restoration of a functional protein, because a non-wild-type amino acid can be incorporated at the PTC position. The human LEPR-b could be more sensitive to the insertion of a non-tryptophan amino acid at codon position 31 than the mouse orthologue. However, recent experiments in yeast showed that UGA codons are predominantly suppressed by tryptophan^58^. Besides, the N-terminal domain (AA residues 22–121) is not crucial for LEPR-b signalling^59, 60^. Therefore, it is more plausible that the broader sequence context in the full-length *hLEPR*-*b* ^*W31X*^ ORF contains nucleotides disturbs translational read-through. The impact of the species backbone on W31X introduces the perspective to discover unknown sequence elements influencing nonsense suppression.

Since the clinical use of aminoglycosides is limited due to cytotoxic effects, other read-through drugs are on demand. One such candidate is the oxadiazole compound PTC124. Best read-through effects of PTC124 are reported on the UGA triplet, especially when a pyrimidine is located in the +1 position^24^. We tested the effect of PTC124 on mutations causing premature UGA codons: *Lepr* ^*W31X*^ (UGA/A), *RLuc* ^*W121X*^ (UGA/G), *RLuc* ^*W156X*^ (UGA/C), *secNLuc* ^*W40X*^ (UGA/C) and *secNLuc* ^*W162X*^ (UGA/A). From this perspective, all mutants – in particular *RLuc* ^*W156X*^ and *secNLuc* ^*W40X*^ – were predicted as PTC124 responders. However, PTC124 induced no restoration of receptor signalling in *mLepr*-*b* ^*W31X*^ transfected cells. The experiments with the nonsense mutated *RLuc* and *secNLuc* reporter genes additionally rebutted a read-through activity of PTC124. Notably, there are preclinical studies which do^24, 26, 28, 29^ or do not^36–38, 40^ substantiate the read-through effect of PTC124. These inconsistencies may be due to differences in the tested nonsense alleles (nature of the PTC and its surrounding context), the selected doses and/or the experimental settings (as suggested by ref. 61). It is a paradox that all 5 tested nonsense alleles were non-responsive to PTC124 but susceptible to aminoglycoside mediated restoration. Critical experimental conditions – for instance concentration range^24, 28–30^, transient transfection^29, 30^, incubation time^24, 28, 29^ and cell line^24, 29^ - match with reports in which PTC124 act as nonsense suppressor. Inconsistent outcomes between studies highlight the need to further characterize PTC124.

In the present study, we investigated the susceptibilities of three LEPR nonsense mutations to stop suppression. The human W31X, mouse Y333X and rat Y763X were all prone to aminoglycoside mediated nonsense suppression when characterized within the mouse receptor. The findings for Y333X and Y763X suggest the corresponding rodent models to explore nonsense suppression *in vivo*. Surprisingly, when the W31X mutation was inserted into its natural human receptor context, its superior read-through susceptibility, observed within the murine sequence, disappeared. The strong impact of the species backbone on W31X provides the possibility to identify novel sequence elements affecting nonsense suppression. The oxadiazole compound PTC124 failed to suppress UGA termination codons, substantiating the need for alternative read-through agents. Our newly designed *Renilla* and *secNLuc* nonsense constructs are suitable as reporters to screen for such alternative compounds.

## Methods

### Generation of Lepr fragment Photinus luciferase fusion constructs for the read-through assay

The Lepr-PLuc fusion constructs consisted of 15 bp nucleotide fragments of the leptin receptor inserted in frame directly after the ATG initiation codon of the *Photinus* luciferase (PLuc) gene in the pGL3-SV40-Promotor vector (Promega, Mannheim, Germany). The receptor fragment comprised the particular PTC ± 6 bp nucleotide context (Table 1 and Supplementary Fig. 1). Fusion constructs were generated by using the QuickChange II site-directed mutagenesis kit (Agilent, Waldbronn, Germany) with oligonucleotides including overhangs encoding the receptor fragments (primer pairs 1–4 in Supplementary Table 1).

### Overexpression and reporter gene vectors for the signalling assay

*mLepr*-*b* ^*wt*^ (UniProt ID P48356) in pcDNA3.1 vector was kindly provided by Dr. Björback. The nonsense mutations W31X, Y333X and Y763X were integrated into the *mLepr*-*b* open reading frame (ORF) using the QuickChange II site-directed mutagenesis kit (Agilent) in accordance to the manufacturer’s instructions. The same mutagenesis strategy was used to insert the W31X mutation in the human *LEPR*-*b* ORF. *hLEPR*-*b* ^*wt*^ (UniProt ID P48357) contained an N-terminal His_6_-tag in pDEST26 (Source BioScience, Nottingham, UK) (Primer pairs 5–8 in Supplementary Table 2). PTP1B overexpression vector was kindly provided by Dr. Liangyou Rui^62^. The STAT3-RE-PLuc reporter gene vector – originally named pAD32 - containing the STAT3-responsive *Photinus* luciferase was also received from Dr. Bjorbaek. Since PTC124 has an off-target effect on the *Photinus* luciferase (PLuc)^38, 39^, we cloned an additional STAT3-RE-RLuc reporter gene vector harbouring the *Renilla* luciferase (RLuc). Therefore, the promotor region from pAD32 containing the STAT3-RE was amplified by PCR. The 336 bp PCR product was cloned with *NheI* and *NcoI* into phRG-b to obtain STAT3-RE-RLuc (primers pair 9 in Supplementary Table 1).

### Generation of nonsense mutated RLuc^W121X^, RLuc^W156X^, secNLuc^W40X^ and secNLuc^W162X^ luciferase reporters

The tryptophan codons (TGG) 121 and 156 in *RLuc* gene in phRG-b (Promega) were replaced by premature TGA stop codons utilizing the QuickChange II mutagenesis kit (Agilent) (Primer pairs 10 and 11 in Supplementary Table 1). The ORF of the secretory NanoLuc luciferase (secNLuc) was amplified by PCR using pNL1.3 (Promega) as a template (Primer pair 12 in Supplementary Table 1). The 618 bp PCR product was cloned with *AflII* and *XhoI* into pcDNA5/FRT/TO (Life Technologies, Carlsbad, California). Then the TGG codons 40 and 162 of secNLuc were changed to TGA by site-directed mutagenesis (Primer pairs 13 and 14 in Supplementary Table 1).

### Culture conditions and transient transfections of HEK293 cells

HEK293 cells were cultured in DMEM (Sigma Aldrich, Taufkirchen, Germany) containing 10% (v/v) FBS (Biochrom, Berlin, Germany) and 200 U/mL penicillin (Carl Roth, Karlsruhe, Germany). One day prior to calcium phosphate transfection, cells from one 10 cm dish were split 1:5 onto new 10 cm dishes (details see ref. 53). Specific procedures for every sub-experiment after transfection are described below:

(i) Read-though assay with ‘Lepr-PLuc fusion constructs’: To test whether the leptin receptor nonsense mutations are prone to aminoglycoside mediated nonsense suppression, HEK293 cells were co-transfected with one of the Lepr-PLuc fusion constructs along with phRG-b vector (5 µg of each plasmid). One day after transfection, cells were transferred from one 10 cm dish to one poly-D-lysine coated 48-well culture plate. Two days following transfection, cells were incubated for 24 h with or without G418 (Sigma Aldrich) or gentamicin (Carl Roth). Three days after transfection, cells were washed with PBS and stored at −80 °C.
(ii) Signalling assay in the presence of aminoglycosides: For assessing the signalling properties of full-length LEPR-b variants in the presence of aminoglycosides, HEK293 cells were co-transfected with three different plasmids (5 µg each): one of the *mLepr*-*b*-pcDNA3.1 expression construct, STAT3-RE-PLuc and phRG-b. One day following the transfection, cells were transferred from one 10 cm dish to one poly-D-lysine coated 48-well plate. Two days after the transfection, gentamicin or G418 were added to the cell cultures. After 6 h pre-incubation with the aminoglycosides – cells were treated with murine leptin containing an N-terminal His_6_-tag (kindly provided by Dr. Martin Schlapschy). Overall, aminoglycoside incubation took 24 h, whereas leptin treatment lasted 18 h. For cells transfected with *hLEPR*-*b*-pDEST26 constructs, aminoglycoside pre-incubation started simultaneously with the transfer to the 48-well plate to allow a treatment time of 48 h. Incubation with human leptin (R&D systems, Minneapolis, MN) lasted also 18 h. Three days after transfection, cells were washed with PBS and stored at −80 °C.
(iii) Signalling assay in the presence of PTC124: For investigating the signalling properties of murine Lepr-b^W31X^ in the presence of PTC124 (Selleckchem Co, Shanghai, China) or G418, HEK293 cells were co-transfected with two different plasmids (5 µg each): *mLepr*-*b* ^*wt*^ or *mLepr*-*b* ^*W31X*^-pcDNA3.1 expression construct along with STAT3-RE-RLuc reporter gene vector. One day post transfection, cells were transferred from one 10 cm dish to one poly-D-lysine 48-well plate. Two days after the transfection, PTC124 or G418 were added to the cells. Three days post transfection, cells were washed with PBS and stored at −80 °C.
(iv) Read-through assay with *RLuc* variants: HEK293 cells were transfected with expression constructs carrying either *RLuc* ^*W121X*^ or *RLuc* ^*W156X*^. One day following transfection, cells were transferred from one 10 cm dish to one poly-D-lysine coated 48-well plate. Two days after the transfection, aminoglycosides or PTC124 were added to the cells. After 24 and 48 h incubation time, cells were washed with PBS and stored at −80 °C.
(v) Read-through assay with *secNLuc* variants: HEK293 cells were transfected with expression constructs carrying either *secNLuc* ^*W40X*^ or *secNLuc* ^*W162X*^. One day post transfection, cells were transferred from one 10 cm dish to one poly-D-lysine coated 48-well plate. Two days after the transfection, G418 or PTC124 were added to the cells. Medium from cells expressing secNLuc^W40X^ or secNLuc^W162X^ was collected 24 and 48 h after the addition of G418 or PTC124. Seventy-two hours after the incubation started, cells were washed with PBS and stored at −80 °C. secNLuc activities were assessed in the medium and in cell lysates.

### Luciferase assays

Quantification of luciferase activities were performed with commercially available kits (Promega, Mannheim, Germany) in concordance to the manufacturer’s instructions. Frozen cells from sub-experiments (i), (ii), (iii) and (iv) were incubated with the passive lysis buffer enclosed in the respective luciferase assay kit for 20 min at room temperature. Lysates from (i) and (ii) were combined with dual luciferase assay reagents (Promega, #E1910), whereas lysates from (iii) and (iv) were mixed with the *Renilla* assay reagent (Promega, #E2810). Bioluminescence was measured in a Sirius single-tube luminometer (Berthold Technologies, Bad Wildbad, Germany). In (i) and (ii) PLuc activities were normalized to those of the constitutively expressed RLuc enzyme derived from phRG-b vector. Medium and cells collected in experiment (v) were processed with compounds from the Nano Glo assay kit (Promega, #N1110) in accordance to the manufacturer’s instructions. secNLuc bioluminescence was quantified in an Infinite M200 plate reader (Tecan, Männedorf, Switzerland).

### Statistics

Data are plotted as means with standard deviations (SD) and were statistically analysed by Prism 6 (Graph Pad software company) and Sigmaplot 12.5 (Systat Software, Erkrath, Germany). Data from the read-through assays obtained with the *Lepr*-*PLuc*, *RLuc* and *secNLuc* reporter genes were analysed by one-way ANOVA with read-through compound concentration as an independent variable. To analyse the data from the signalling assays, two tailed t-tests were conceded to test for significant differences between leptin (+) and non-leptin (−) treated cells within each aminoglycoside/PTC124 concentration. Statistical significances were corrected for multiple comparisons by applying the Holm-Sidak method. Data from the hLEPR-b signalling assay were additionally analysed by two-way ANOVA with aminoglycoside and leptin as independent variables.

## Electronic supplementary material

Supplements
