# Supplementary material for: Aminoglycosides, but not PTC124 (Ataluren), rescue nonsense mutations in the leptin receptor and in luciferase reporter genes
Source: Sci Rep. 2017 Apr 21;7:1020. doi: 10.1038/s41598-017-01093-9 (PMC5430635; doi:10.1038/s41598-017-01093-9)
Supplement: Supplementary file 1 — Supplements [file 41598_2017_1093_MOESM1_ESM.pdf]

Supplementary Information

Aminoglycosides, but not PTC124 (Ataluren), rescue nonsense mutations in the leptin receptor and in luciferase reporter genes

Florian Bolze, Sabine Mocek, Anika Zimmermann and Martin Klingenspor*

ZIEL - Institute for Food and Health, Technical University of Munich, Gregor-Mendel-Str. 2, Freising, Germany *and* Chair of Molecular Nutritional Medicine, Technical University of Munich, EKFZ - Else Kröner-Fresenius-Center for Nutritional Medicine, Gregor-Mendel-Str. 2, Freising, Germany

*To whom correspondence should be addressed: Chair of Molecular Nutritional Medicine, Technische Universität München, Gregor-Mendel-Straße 2, Freising, Germany, Fax: +49 8161 71 2404, E-mail: mk@tum.de (M.Klingenspor)

1. Supplementary Table

Supplementary Table 1. Oligonucleotides. *Primer pairs 1-4*: nucleotides in bold indicate the overhang encoding the leptin receptor fragment. The PTCs are underlined. *Primer pairs 5-11*: Nucleotides in bold indicate mutated positions. Primer pairs 5 and 6 contain synonymous mutations for *HindIII* and *BglII* restriction sites, respectively. Underlined bases highlight the PTC except for pairs 9, 12 and 15 – here the restriction sites for cloning are indicated.

Pair	Construct		Primer sequence
1	*mLepr*^{W31X}*-PLuc*	For<br>Rev	**GAAAATTT**GAAGACGCCAAAAACATAAAGAAAGGCCCGGCGCCATTCTATC<br>**AGGGAGA**CATGGTGGCTTTACCAACAGTACCGGAATGCCAAGC
2	*mLepr*^{Y333X}*-PLuc*	For<br>Rev	**AATTTCCA**GAAGACGCCAAAAACATAAAGAAAGGCCCGGCGCCATTCTATC<br>**ACACAAC**CATGGTGGCTTTACCAACAGTACCGGAATGCCAAGC
3	*mLepr*^{Y763X}*-PLuc*	For<br>Rev	**AAAGTCTG**GAAGACGCCAAAAACATAAAGAAAGGCCCGGCGCCATTCTATC<br>**AATCATC**CATGGTGGCTTTACCAACAGTACCGGAATGCCAAGC
4	*hLEPR*^{W31X}*-PLuc*	For<br>Rev	**GAAGATTT**GAAGACGCCAAAAACATAAAGAAAGGCCCGGCGCCATTCTATC<br>**AAGGAGT**CATGGTGGCTTTACCAACAGTACCGGAATGCCAAGC
5	*mLepr-b*^{W31X}	For<br>Rev	CCAATCTCTCCCTG**A**AAATTTAAG**C**T**T**TTTTGTGGACCACCG<br>CGGTGGTCCACAAAA**A**A**G**CTTAAATTT**T**CAGGGAGAGATTGG
6	*mLepr-b*^{Y333X}	For<br>Rev	CAAGATGTTGTGTA**A**TTTCCACCCAA**G**AT**CT**TGACTAGTG<br>CACTAGTCA**AG**AT**C**TTGGGTGGAAA**TTA**CACAACATCTTG
7	*mLepr-b*^{Y763X}	For<br>Rev	GTCACCTGATGATTA**A**AGTCTGTTATATCTGG<br>CCAGATATAACAGACT**T**TAATCATCAGGTGAC
8	*hLEPR-b*^{W31X}	For<br>Rev	TGTCATATCCAATTACTCCTTG**A**AGATTTAAGTTGTCTTGCATGC<br>GCATGCAAGACAACTTAAATCT**T**CAAGGAGTAATTGGATATGACA
9	*STAT3-RE-phRG-b*	For<br>Rev	CACGCTAGCTGAATGCAATTGTTGTTGTTAACTTG<br>ACCCATGGTACCAACAGTACCGGAATGCC
10	*RLuc*^{W121X}	For<br>Rev	GGGCCACGACTG**A**GGGGCTTGTCTG<br>CAGACAAGCCCC**T**CAGTCGTGGCCC
11	*RLuc*^{W156X}	For<br>Rev	CCTGGGACGAGTG**A**CCTGACATCGAGG<br>CCTCGATGTCAGG**T**CACTCGTCCCAGG
12	*secNLuc ORF*	For<br>Rev	CTTAGGGCCACCATGAACTCCTTCTCCACAAGCGCCTT<br>CTCGAGTTACGCCAGAATGCGTTCGCACAGC
13	*secNLuc*^{W40X}	For<br>Rev	TCGTTGGGGACTG**A**CGACAGACAGCCG<br>CGGCTGTCTGTCG**T**CAGTCCCCAACGA
14	*secNLuc*^{W162X}	For<br>Rev	TGTAACAGGGACCCTGTG**A**AACGGCAACAAAATTATC<br>GATAATTTTGTTGCCGTT**T**CACAGGGTCCCTGTTACA
15	*hLEPR-b ORF*	For<br>Rev	GCGGCCGCACCATGATTTGTCAAAAATTCTGTG<br>AAGCTTTTACACAGTTAGGTCACACATCTTGTT

2. Supplementary Figures

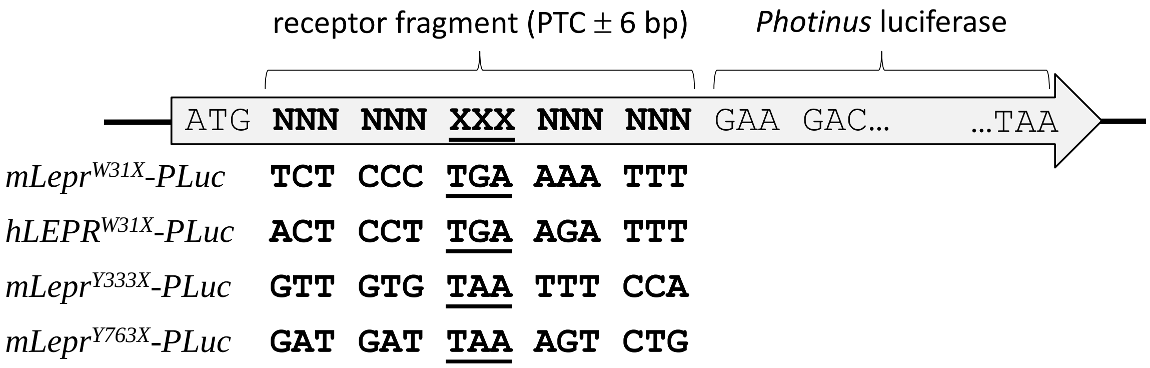

Supplementary Fig. 1. Structure of LEPR-PLuc reporter fusion constructs. The reporter constructs consisted of a 15 bp fragment of the leptin receptor gene and the full-length *Photinus* luciferase (PLuc). The premature termination codons (XXX) from W31X, Y333X and Y763X including ± 6 bp sequence context (± NNN NNN) were fused upstream of the coding sequence of *PLuc*.

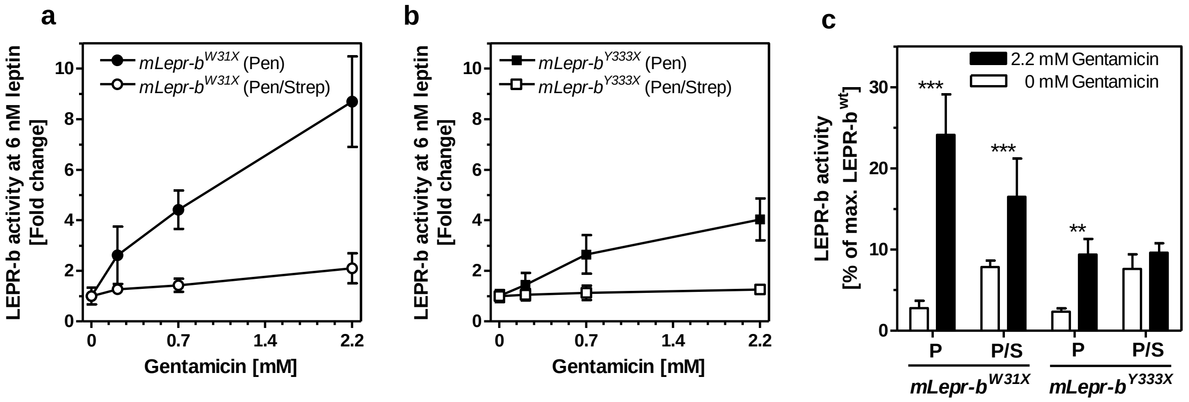

Supplementary Fig. 2. Streptomycin in the cell culture medium lowers the efficacy of gentamicin to rescue mLEPR-b^{W31X} and mLEPR-b^{Y333X} signalling. HEK293 cells were co-transfected with either *mLepr-b^{W31X}* or *mLepr-b^{Y333X}*-pcDNA3.1, STAT3-RE-PLuc and phRG-b. Cells were maintained in medium with penicillin (P) or with penicillin/streptomycin (P/S). Two days after the transfection, cells were treated for 24 h with rising concentrations of gentamicin and for the last 16 h in addition with 6 nM murine leptin (n=4, SD). Data in **(a)** and **(b)** are expressed in fold change relative to the receptor activity level without gentamicin (0 mM). In **(c)**, data are shown in percent relative to the receptor activity measured in cells transfected with *Lepr-b^{wt}*-pcDNA3.1 after stimulation with 6nM leptin. In **(d),** statistical significance between 0 and 2.2 mM gentamicin for each condition was assessed by t-tests with Bonferroni-Holm correction for multiple comparisons **p<0.01; ***p<0.001.

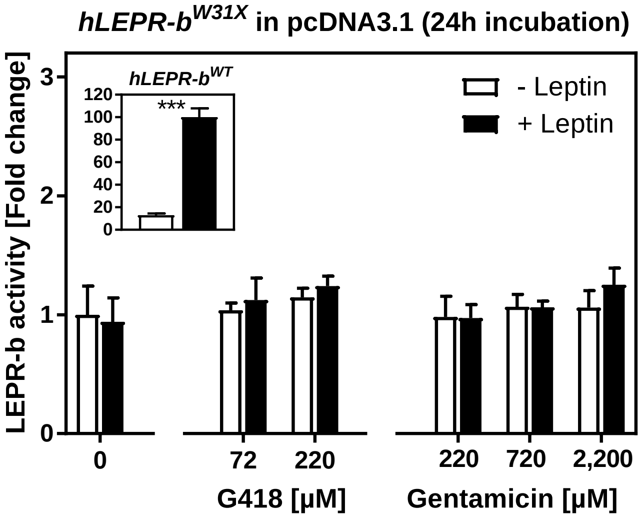

Supplementary Fig. 3. G418 and gentamicin did not rescue hLEPR-b^{W31X} signalling when expressed from pcDNA3.1 vector. HEK293 cells were co-transfected with either the *hLEPR-b*^{W31X}- (Inset) or *hLEPR-b*^{WT} pcDNA3.1 overexpression construct, STAT3-RE-PLuc and phRG-b. Two days after the transfection, cells were treated for 24 h with the indicated concentrations of gentamicin or G418 and for the last 16 h in addition with 12.5 nM human leptin (n=3, SD). Statistical significance was assessed by t-tests with

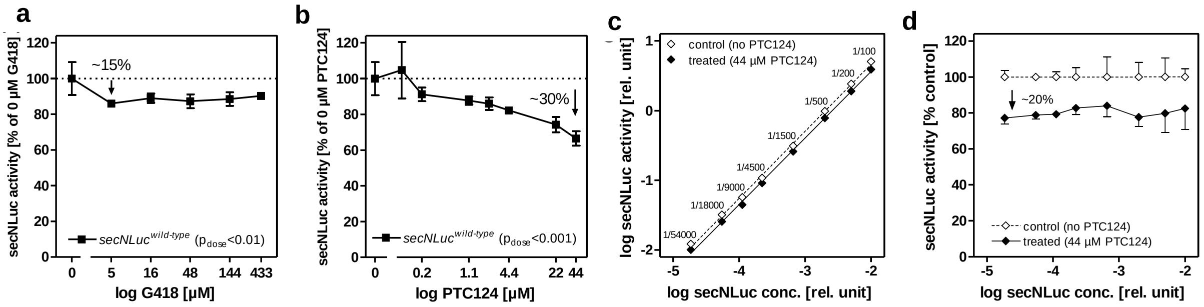

Supplementary Fig. 4. Effect of G418 and PTC124 on secNLuc$^{wild\text{-}type}$ activity during luminometry. Medium from HEK293 cells transiently expressing *secNLuc$^{wild\text{-}type}$* was collected, mixed with NLuc assay buffer and supplemented with rising concentrations of either **(a)** G418 or **(b)** PTC124. secNLuc$^{wild\text{-}type}$ activities were quantified in a luminometer (n=4, SD). Medium containing secNLuc$^{wild\text{-}type}$ was diluted, mixed with assay buffer and constantly supplemented with 44 µM PTC124 **(c)** or **(d)**. Fractions in (c) indicate dilution steps (n=4, SD).

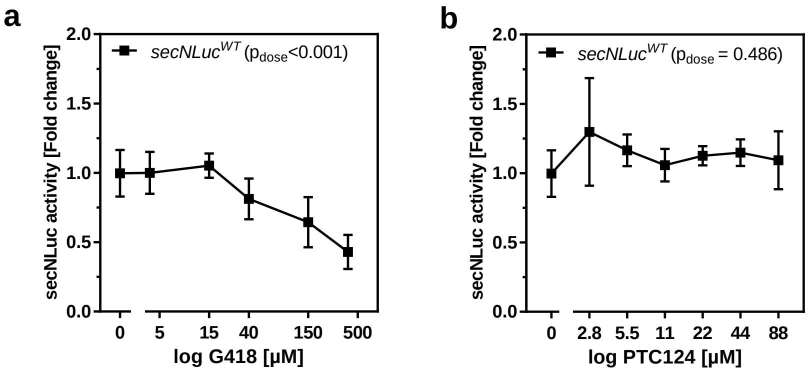

Supplementary Fig. 5. Effect of G418 and PTC124 on secNLuc$^{wild\text{-}type}$ expression. HEK293 cells were transiently transfected with the *secNLuc$^{wild\text{-}type}$* overexpression construct. Two days after the transfection, cells were treated for 24 h with rising concentrations of **(a)** G418 or **(b)** PTC124**.** Cells were washed and luciferase activity was measured in lysates. P-values indicate the outcome from one-way ANOVA with the dose of the test compound as independent variable (n=4, SD).

3. Supplementary Results and Discussion

The aminoglycoside antibiotic streptomycin lowers the efficacy of gentamicin to rescue mLEPR-b^{W31X} and mLEPR-b^{Y333X} signalling. Standard cell culture medium is often supplemented with penicillin and streptomycin to prevent contaminations. Here, we performed the signalling assay with medium containing 100 U/ml penicillin and 100 µg/ml (170 µM) streptomycin (Biochrom) or medium only supplemented with 200 U/ml penicillin.

The presence streptomycin reduced the efficiency of gentamicin to rescue mLEPR-b^{W31X} and mLEPR-b^{Y333X} signalling in HEK293 cells. When comparing the receptor activities measured in cells incubated with 6 nM leptin, only a doubling of mLEPR-b^{W31X} signalling between 0 vs. 2.2 mM gentamicin was observed when medium contained streptomycin (Supplementary Fig. 2 a). Without streptomycin, the gentamicin effect on mLEPR-b^{W31X} signalling at 6 nM leptin was more than 8-fold (Supplementary Fig. 2 a).

Streptomycin completely blocked the rescue effect of gentamicin on mLEPR-b^{Y333X} (Supplementary Fig. 2 b). Only in the absence of streptomycin, gentamicin revived mLEPR-b^{Y333X} activity (Supplementary Fig. 2 b). This outcome can be explained by the fact that medium with streptomycin might promote 'basal' read-through at PTCs (Supplementary Fig. 2 c). Additionally, streptomycin could compete with gentamicin for the ribosomal binding site and thereby impairs the effect of gentamicin (Supplementary Fig. 2 c). This outcome demonstrates the importance of optimal cell culture conditions to explore the activity of nonsense suppressor compounds.

G418 and gentamicin do not to rescue hLEPR-b^{W31X} when expressed from pcDNA3.1. Mouse and human full-length LEPR-b were expressed from different vector backbones (pcDNA3.1 vs. pDEST26) (Fig. 4). To align the expression system, we transferred *hLEPR-b^{wt}* and *hLEPR-b^{W31X}* into pcDNA3.1. The respective human ORFs - without the N-terminal His_6-tag - were PCR amplified using the pDEST26 constructs as templates (primer pair 15 in Supplementary Table 1). The 3512 bp PCR products were cloned into pcDNA3.1 using the restriction enzymes *NotI* and *HindIII*. The functional efficiency of the pcDNA3.1 expression constructs was confirmed by the activation of hLEPR-b^{WT} (inset Supplementary

Fig. 3). The expression of *hLEPR-b^{W31X}* from pcDNA3.1 was not changing the susceptibility to nonsense suppression in comparison to the pDEST26 constructs (Fig. 4) since G418 and gentamicin did not rescue hLEPR-b^{W31X} activity (Supplementary Fig. 3).

Effect of G418 and PTC124 on secNLuc$^{wild\text{-}type}$. To explore whether G418 and PTC124 have off-target effects on secNLuc, we cloned *secNLuc$^{wild\text{-}type}$* ORF into pcDNA5/FRT/TO. In the first place, we tested whether G418 and PTC124 disturb the bioluminescence reaction catalysed by secNLuc$^{wild\text{-}type}$. To do so, medium from HEK293 cells transiently expressing secNLuc$^{wild\text{-}type}$ was collected and 500-fold diluted in water. Ten µl of this dilution were mixed with 10 µl NLuc assay buffer supplemented with raising amounts of G418 and PTC124 in 1 µl. Both compounds triggered a significant reduction of the bioluminescence signal indicating an inhibitory effect on secNLuc activity especially in the presence of PTC124 (Supplementary Fig. 4 a and b). To further characterize the effect of PTC124 on the bioluminescence reaction, we added a constant concentration of 44 µM PTC124 to different amounts of secNLuc$^{wild\text{-}type}$. Therefore, medium containing secNLuc protein was diluted (100- to 54,000-fold) and mixed with 44 µM PTC124. Independent from the secNLuc concentration, 44 µM PTC124 caused a consistent ~20% depression of the luminescence signal (Supplementary Fig. 4 c and d).

In the next step, we tested whether G418 and PTC124 affect expression of secNLuc$^{wild\text{-}type}$ in transiently transfected HEK293 cells (e.g. via influencing transcription, transcript/protein stability or toxicity). Medium was supplemented with rising concentrations of G418 of PTC124. After 24 h incubation, cells were washed with PBS and lysed with 20 µl NLuc assay buffer and 20 µl water. This lysate was 10-fold diluted in water and 1 µl of this mixture was added to 19 µl NLuc assay buffer to quantify luminescence. We observed that PTC124 had no effect on secNLuc$^{wild\text{-}type}$ expression, whereas G418 induced a significant reduction most likely due to its toxic properties (Supplementary Fig. 5 a and b).

One may speculate that the inhibitory activity of PTC124 on the bioluminescence reaction (Supplementary Fig. 4) could mask a translational read-through effect. The consistent ~20% depression of the bioluminescence signal over a large secNLuc$^{wild\text{-}type}$ concentration range illustrates that only a

modest read-through activity of the same effect strength would be masked by PTC124s off-target effect during luminometry. Thus, we evaluate secNLuc as suitable reporter to study nonsense suppression.
